# The HIV-1 Nucleocapsid Regulates Its Own Condensation by Phase-Separated Activity-Enhancing Sequestration of the Viral Protease during Maturation

**DOI:** 10.3390/v13112312

**Published:** 2021-11-19

**Authors:** Sébastien Lyonnais, S. Kashif Sadiq, Cristina Lorca-Oró, Laure Dufau, Sara Nieto-Marquez, Tuixent Escribà, Natalia Gabrielli, Xiao Tan, Mohamed Ouizougun-Oubari, Josephine Okoronkwo, Michèle Reboud-Ravaux, José Maria Gatell, Roland Marquet, Jean-Christophe Paillart, Andreas Meyerhans, Carine Tisné, Robert J. Gorelick, Gilles Mirambeau

**Affiliations:** 1 Infectious Disease & AIDS Research Unit, Institut d’Investigacions Biomèdiques August Pi i Sunyer (IDIBAPS), Villaroel 170, 08036 Barcelona, Spain; cristina.lorca.oro@gmail.com (C.L.-O.); sara.nietoma@gmail.com (S.N.-M.); tuixent.escriba@gmail.com (T.E.); natgabrielli@gmail.com (N.G.); xiao-tan@hotmail.com (X.T.); mohamed.ouizougun.oubari@gmail.com (M.O.-O.); jose.okoronkwo@gmail.com (J.O.); josemaria.x.gatell@viivhealthcare.com (J.M.G.); 2 Centre d’Etudes des Maladies Infectieuses et Pharmacologie Anti-Infectieuse (CEMIPAI), CNRS UAR 3725, Université de Montpellier, 1919 Route de Mende, CEDEX 05, 34293 Montpellier, France; 3 Infection Biology Laboratory, Department of Experimental and Health Sciences (DCEXS), Universitat Pompeu Fabra, Carrer Doctor Aiguader 88, 08003 Barcelona, Spain; Andreas.Meyerhans@upf.edu; 4 Molecular and Cellular Modeling Group, Heidelberg Institute for Theoretical Studies (HITS), Schloss-Wolfsbrunnenweg 35, 69118 Heidelberg, Germany; 5 Genome Biology Unit, European Molecular Biology Laboratory, Meyerhofstrasse 1, 69117 Heidelberg, Germany; 6 Biological Adaptation and Ageing (B2A), CNRS UMR 8256 & INSERM ERL U1164, Institut de Biologie Paris-Seine (IBPS), Faculté des Sciences et d’Ingénierie (FSI), Sorbonne Université, 7 Quai St Bernard, CEDEX 05, 75252 Paris, France; dufaulaure@gmail.com (L.D.); michele.reboud@upmc.fr (M.R.-R.); 7 Facultat de Medicina y Ciencias de la Salud, Universitat de Barcelona, Carrer de Casanova 143, 08036 Barcelona, Spain; 8 Architecture et Réactivité de l’ARN, CNRS UPR 9002, Université de Strasbourg, 2 Allée Conrad Roentgen, 67000 Strasbourg, France; r.marquet@ibmc-cnrs.unistra.fr (R.M.); jc.paillart@ibmc-cnrs.unistra.fr (J.-C.P.); 9 Institució Catalana de Recerca i Estudis Avançats (ICREA), Passeig de Lluís Companys 23, 08010 Barcelona, Spain; 10 Expression Génétique Microbienne, CNRS UMR 8261, Institut de Biologie Physico-Chimique (IBPC), Université de Paris, 13 Rue Pierre et Marie Curie, 75005 Paris, France; carine.tisne@ibpc.fr; 11 AIDS and Cancer Virus Program, Leidos Biomedical Research, Inc., Frederick National Laboratory for Cancer Research, Frederick, MD 21701, USA; gorelicr@mail.nih.gov; 12 Biologie Intégrative des Organismes Marins (BIOM), CNRS UMR 7232, Observatoire Océanologique de Banyuls (OOB), Faculté des Sciences et d’Ingénierie (FSI), Sorbonne Université, 1 Avenue Pierre Fabre, 66650 Banyuls-sur-Mer, France

**Keywords:** HIV-1, nucleocapsid, RNA, liquid–liquid phase separation, protease, molecular dynamics, atomic-force microscopy, biomolecular condensates, enzyme catalysis

## Abstract

A growing number of studies indicate that mRNAs and long ncRNAs can affect protein populations by assembling dynamic ribonucleoprotein (RNP) granules. These phase-separated molecular ‘sponges’, stabilized by quinary (transient and weak) interactions, control proteins involved in numerous biological functions. Retroviruses such as HIV-1 form by self-assembly when their genomic RNA (gRNA) traps Gag and GagPol polyprotein precursors. Infectivity requires extracellular budding of the particle followed by maturation, an ordered processing of ∼2400 Gag and ∼120 GagPol by the viral protease (PR). This leads to a condensed gRNA-NCp7 nucleocapsid and a CAp24-self-assembled capsid surrounding the RNP. The choreography by which all of these components dynamically interact during virus maturation is one of the missing milestones to fully depict the HIV life cycle. Here, we describe how HIV-1 has evolved a dynamic RNP granule with successive weak–strong–moderate quinary NC-gRNA networks during the sequential processing of the GagNC domain. We also reveal two palindromic RNA-binding triads on NC, KxxFxxQ and QxxFxxK, that provide quinary NC-gRNA interactions. Consequently, the nucleocapsid complex appears properly aggregated for capsid reassembly and reverse transcription, mandatory processes for viral infectivity. We show that PR is sequestered within this RNP and drives its maturation/condensation within minutes, this process being most effective at the end of budding. We anticipate such findings will stimulate further investigations of quinary interactions and emergent mechanisms in crowded environments throughout the wide and growing array of RNP granules.

## 1. Introduction

Biomolecular condensates (BCs) are membraneless, intracellular assemblies formed by the phenomenon of liquid–liquid phase separation (LLPS) [1,2,3,4,5]. Several types of such assemblies have been observed inside eukaryotes with a variety of suggested functions. These range from adaptive cellular responses to physiological stresses via the formation of stress granules [6,7,8,9] to meeting the demands of intracellular transport or signalling, amongst many other functions [3]. They have also importantly been linked to disease [10,11]. Fundamentally, due to their capacity to concentrate biomolecules, a suggested principal function of BCs has been that they regulate enzyme biochemistry [12,13,14,15,16]. Many condensates sequester mRNAs and associated RNA-binding proteins into what are termed RNA granules [17,18,19,20,21,22,23,24]. The material properties of such granules can vary depending on composition and biological functionality [25]—from dynamic architectures with liquid-like phases to non-dynamic gel-like phases [26]. Phase transitions between liquid- to gel-like phases due to condensate ageing have also been observed [27].

The concept of quinary interactions [28,29]—the emergent sum of many transient weak interactions that may occur in a crowded biomolecular environment—has been suggested to promote the assembly of highly stable but dynamic and changing multi-macromolecular complexes without any requirement for membrane compartmentalisation [30,31,32,33,34]. Compatible with this concept, multivalent molecules that enable the assembly of dense networks of weak interactions are emerging as major molecular drivers that underpin the formation of BCs [35,36,37,38]. In particular, the cooperation between long polymers, such as RNAs, together with folded proteins and intrinsically disordered proteins (IDPs) may be an essential feature of many condensates [3,39,40]. Furthermore, constituent binding affinity, valency, liquid network connectivity, and critical post-translational modifications all play a role in regulating BCs [41,42,43,44,45,46,47,48].

Recently, constituents of RNA-containing viruses, such as HIV-1 and SARS-CoV-2, have been shown to phase-separate into biomolecular condensates inside cells [49], using their repertoire of IDPs [50] in conjunction with the RNA-binding capacity of their nucleocapsid proteins to interact with genomic RNA (gRNA) elements [51,52,53,54,55,56].

**Figure 1:**
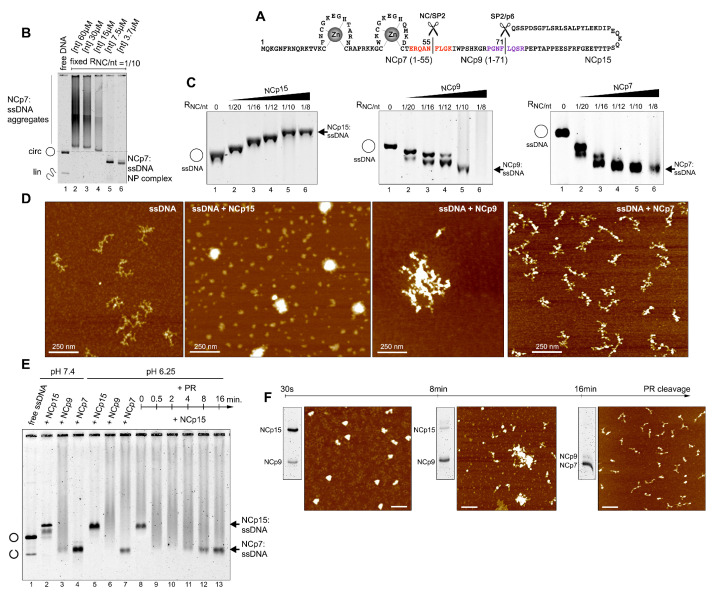
**Quinary interactions and architectural behaviour of NC:NA NP complexes upon PR processing.** (**A**) Sequence of NCp15 and PR cleavage sites (NC/SP2 in red, SP2/P6 in magenta). (**B**) Electrophoretic migration on agarose gel of M13 ssDNA:NCp7 NP complexes. Serial dilutions of both ssDNA (expressed in total [nt]) and NCp7, keeping a constant ratio RNC/nt = 1/10, were incubated for 30 min at 37 ${}^{\circ }$C before electrophoresis at 4 ${}^{\circ }$C and DNA staining. DNA smears; hallmarks of ssDNA/NCp7 aggregates (lanes 2–4) are converted into fast migrating ssDNA/NCp7 condensates (lanes 5–6) upon dilution. Position of circular ssDNA and traces of linear ssDNA are indicated. (**C**) Comparison of the electrophoretic mobility in 1% agarose of the circular M13 ssDNA in complex with NCp15, NCp9, and NCp7 upon increasing protein concentration. RNC/nt indicate the protein/nucleotide ratio. (**D**) AFM imaging of M13 ssDNA (0.4 nM) incubated from left to right without protein, with NCp15, NCp9, and NCp7 at RNC/nt = 1/10. (**E**,**F**) Sequential proteolysis of M13 ssDNA-bound NCp15 followed by EMSA, SDS-PAGE, and AFM. ssDNA (1 nM):NCp15 (750 nM) complexes were assembled at pH 6.25 and subsequently cleaved by PR (35 nM) at the indicated times at 37 ${}^{\circ }$C. Each reaction was stopped by chilling the tubes on ice, and the tube contents were used to follow ssDNA:NC complex migration by EMSA (E), the maturation state of NC proteins by SDS-PAGE (inserts in F), and ssDNA:NC complex morphology by AFM (F) at t = 30 s, 8 min and 16 min. In (E), NC:NA complex migration controls for NCp15, NCp9, and NCp7 are shown at pH 7.4 and pH 6.25 (optimal for PR cleavage).

Even though an HIV-1 particle is derived from the self-assembled Pr55Gag shell and is ultimately enveloped by a lipid membrane, the concept of quinary interactions is clearly applicable in describing its dynamic assembly at the mesoscopic scale since it forms a confined phase-separated RNP in a highly crowded space, within a limited time frame, and in a cooperative manner. Pr55Gag is composed from the N- to C-termini of matrix (MAp17), capsid (CAp24), spacer peptide SP1, nucleocapsid (NC), spacer peptide SP2, and p6 protein. The key players here consist of NC protein intermediates with variable nucleic acid (NA) binding properties that are dependent upon their processing state [57,58,59,60]. Tethered within the virion by approximately 2400 GagNC domains, the two single strands of 9.2 kb-long gRNA specifically scaffold Pr55Gag self-assembly. Subsequently, the HIV-1 RNP complex undergoes a granular condensation during the sequential proteolysis of the Pr55Gag RNA-binding domain into the mature nucleocapsid protein (NCp7) by the viral protease (PR) [59,61,62]. PR is derived by the autoprocessing of a smaller number of GagPol within the Pr55Gag assembly that additionally contain reverse transcriptase (RT) and integrase (IN). Approximately 60 PR homodimers are potentially available to catalyse maturation, which principally requires around 12,000 cleavage events. Cleavage of GagNC by PR first generates NCp15 (NCp7-SP2-p6) bound to, and forming with, the gRNA a ribonucleoprotein (RNP) intermediate that physically detaches from the remaining outer MA-CA-SP1 shell. The second cleavage between SP2 and p6 releases NCp9 (NCp7-SP2) (Figure 1A). Single-stranded nucleic acids (ssNA) stimulate both cleavage events in vitro [58,63,64]. The third cleavage produces the mature 55 amino acids (aa)-long NCp7 and SP2. Within the virus, NCp15 seems to condense gRNA less well than NCp9 and NCp7 [65]. Yet, NCp9 does not appear as functional as NCp7 [66]. NCp15 and NCp9 are short-lived species not detected during typical virus production [60]. Why such intermediates are maintained along the HIV-1 maturation process remains unclear.

HIV-1 PR is an aspartyl-protease, enzymatically active only as a homodimer. Recombinant PR is stabilized in vitro by high ionic strength (>1 M NaCl), and catalysis is strongly activated under acidic conditions (pH 5.0 or even lower). Lower salt (0.1 M NaCl) and increasing the pH to 6.0 limit the acidic catalysis and shift the equilibrium towards the monomer [67]. At quasi-neutral pH, in low salts and an excess of PR, the in vitro cleavage of Gag follows the sequential mechanism described above leading to NCp7 and the condensed RNP [68]. RNA or ssDNA promotes NCp15 cleavage in vitro [58,69], while recent reports have shown that direct RNA-PR contacts enhance the enzyme activity [64]. Consequently, PR appears to engage in an intricate partnership with NC and gRNA during viral maturation that remains incompletely understood. HIV-1 NCp7 contains a small globular domain formed with two zinc fingers (ZFs) that generate a hydrophobic pocket with two aromatic residues (Phe16 and Trp37). This platform stacks with unpaired nucleotides, preferentially guanosines exposed in RNA or ssDNA secondary structures, while basic residues stabilize the complex through electrostatic interactions with the NA backbone. With particular stem-loops in gRNA or DNA, this results in the formation of specific complexes [70,71]. NCp7 is also a highly mobile and flexible polycationic condensing agent; similar to polyamines, transient protein:NA electrostatic contacts neutralize phosphate backbone repulsions, lowering the overall energy of the RNP complex [59,72,73].

The binding properties of the various maturation states of the nucleocapsid protein to nucleic acids vary [74,75,76]. In vitro, these properties induce a massive co-aggregation of recombinant NCp7 and NCp9 with NA templates [57,60,77]. This quinary interaction capability guides the matchmaking/NA chaperone activity by facilitating intra- and intermolecular RNA–RNA interactions required for functional gRNA folding [78]. Such crowding effects rely on basic residues particularly concentrated in the two small flexible domains, the (1–14) N-terminal domain and the (29–35) linker between the ZFs [72]. NCp15 shows slightly different NA binding and chaperone properties but is essentially characterized by a reduced ability to aggregate NA [57,60,79], properties recently correlated with a direct fold-back contact between the p6 and ZF domains [60]. NCp9 shows an enhanced NA affinity due to a slower dissociation rate, as well as dramatically enhanced NA aggregating activities [57,60,73]. Alanine substitution of acidic residues in p6 converts NCp15 to an NA-aggregating protein similar to NCp9, while the addition of a p6 peptide lowers the RNA chaperone activity of NCp7 in vitro [60]. This suggests that SP2 contains an additional NA-interaction domain, which may be masked or modulated with another NCp7 domain by intra- or intermolecular protein contacts between p6 and the NC domain.

HIV-1 maturation is mandatory for viral dissemination following sequential processes of protein and RNA self-assembly, coordinated in space and time by the enzymatic activity of viral PR [61,62,80]. The slow in vitro kinetics of Gag proteolysis supports a general scheme for PR to be auto-processed during the completion of budding, thus driving viral maturation within free, released particles in a computed time-scale close to 30 min [81]. This model is, however, inconsistent with many observations from electron microscopy that show (i) a huge majority of free but freshly released particles in a mature form containing condensed RNP [82], (ii) both capsid and budding defects in the presence of PR inhibitors [83], and (iii) budding and maturation defects for critical NC mutants, whereas Western blots from cell extracts detect PR-processed Gag products [82]. Such findings suggest a much closer overlap between budding and maturation than generally supposed. Importantly, suppressing both PR cleavage sites in NCp15 abolishes viral infectivity [65,84] and results in an abnormal virion core morphology [65]. In contrast, suppression of the NCp7-SP2 cleavage site shows little effect on virus morphology and infectivity in single-cycle assays, but reverts to WT (containing NCp7) after several rounds of infection [84]. A “roadblock” mechanism affecting RT activity on an NA template has been shown to be imparted by NCp9 as well as by NCp15, which could limit large-scale viral replication, highlighting NCp7 as the optimized cofactor for accurate RNP folding and viral fitness [66].

The present study highlights how HIV-1 gRNA becomes condensed by NC proteins through the action of the RNP-sequestered PR enzyme. Reconstituted systems that model non-sequence-specific binding on a large scale, together with molecular dynamics simulations and RNP-modulated enzyme-substrate reaction kinetics theory, allow us (i) to detail the quinary effects and their variations engaged in this dynamic process and (ii) to focus on PR action in such a quinary interaction context.

## 2. Materials and Methods

### 2.1. Proteins, Nucleic Acids, and Reagents

Proteins: The HIV-1 NC proteins and proviral plasmids were based on the pNL4-3 sequence (GenBank accession number AF324493). Recombinant wild-type and mutants of NCp7, NCp9, and NCp15, respectively 55, 71, and 123 amino acids in length, were expressed and purified as described [60,85,86,87]. The CA-SP1-NC-SP2-p6 protein expression construct was generated by PCR amplifying pNL4-3 using GagΔMA sense primer 5${}^{\prime }$-GATCTGGGTACCGAGAACCTCTACTTCCAGATGATAGTGCAGAAC, NL43 OCH antisense primer 5${}^{\prime }$-GCTTGAATTCTTATTGTGACGAGGGGTCGCTGC, and cloning the resulting product into the homologous KpnI and EcoRI sites of pET32a (Novagen, USA). Expression constructs for NCp15(1) (expressing NCp15 that can be cleaved only to NCp9) and NCp15(2) (expressing uncleavable NCp15) were generated starting with the NC-SP2- and NCp15-containing proviral plasmids of Coren et al. [84], respectively. The two constructs were generated by PCR amplifying the appropriate plasmids with NL4-3 NC sense primer 5${}^{\prime }$-CGTGGGATCCTTAGAGAACCTCTACTTCCAGATACAGAAAGGCAATTTTAG, NL4-3 NCp15 antisense primer 5${}^{\prime }$-GTACGTGTCGACTCTCTAATTATTGTGACGAGGGGT CGCT, and cloning into the homologous BamHI and SalI sites of pET32a. Site-directed mutagenesis of the wild-type NL4-3 NCp7 construct to generate the K3A/F6A/Q9A mutant was performed using the Agilent QuickChange Site-Directed Mutagenesis kit (Agilent, USA), with verification by NA sequence analysis, for the generation of the recombinant expression plasmid, as described in [88]. The K3A mutation results from changes to nucleotides 1927 through 1929 from AAA to GCC, F6A results from nucleotides 1936 and 1937 being changed from TT to GC, and Q9A results from nucleotides 1945 and 1946 being changed from CA to GC. Proteins were expressed and purified as described in [60,85,86,87]. Proteins were stored lyophilized and then suspended at a concentration of 1 mg/mL in a buffer containing 20 mM HEPES pH 7.5, 50 mM sodium acetate, 3 mM DTT, 20% (*v*/*v*) ethylene glycol, and 200 μM ZnCl${}_{2}$ and stored at −20 ${}^{\circ }$C. The concentrations were determined by measuring the UV absorbance at 280 nm using the following extinction coefficients: NCp7 and NCp7 mutants: 5690 M${}^{-1}$cm${}^{-1}$; NCp9: 11,380 M${}^{-1}$cm${}^{-1}$; NCp15: 12,660 M${}^{-1}$ cm${}^{-1}$. HIV-1 PR was expressed in E. coli Rosetta(DE3)pLysS strain (Novagen, USA) as inclusion bodies using the expression vector pET-9 and purified as described in [89,90]. The PR domain used here bears the Q7K/L33I/L63I and C67A/C95A protective mutations to respectively minimize autoproteolysis [67] and prevent cysteine-thiol oxidation [91]. PR was suspended, adjusted to 10–20 μM concentration, and stored at −80 ${}^{\circ }$C in 50 mM sodium acetate pH 5.5, 100 mM NaCl, 1 mM DTT, 0.1 mM EDTA, and 10% (*v*/*v*) glycerol.

Nucleic Acids: The circular 7249 nt M13 ssDNA (m13mp18) was purchased (Bayou Biolabs, USA), as was the 3569 nt MS2 RNA (Roche GmBh, Germany). Linear m13mp18 molecules were generated by annealing a complementary oligonucleotide to form a restriction site for BsrB I (NEB, USA) as described in [92]. The oligonucleotides poly d(A)13 and TAR-RNA (27 nt, 5′-CCAGAUCUGAGCCUGGGAGCUCUCUGG-3′) were purchased (Sigma-Aldrich, Germany), the short RNA fragments corresponding to individual stem-loop motifs of the Psi region: SL1 (17 nt), SL2 (23 nt), SL3 (14 nt), and SL4 (24 nt) were purchased (Microsynth, Switzerland) and purified by HPLC (Äkta design-Unicorn, Cytiva, USA) on a PA-100 anion exchange column (Dionex, Thermo Fisher Scientific, USA). Plasmids used for in vitro transcription of HIV-1 RNAs used in this study have been described previously [93,94]. Briefly, the pJCB vector was linearized with AflII, XbaI, BssHII, RsaI, or PvuII and used as templates for the synthesis of RNAs 1–61, 1–152, 1–278, 1–311, and 1–615, respectively, by in vitro run off transcription using bacteriophage T7 RNA polymerase, followed by purification using size exclusion chromatography as described previously [95]. Likewise, plasmid pmCG67 was linearized with AvaII or SalI to produce RNAs 1–1333 and 1–4001, respectively. RNA 1–102 was obtained from a PCR product corresponding to the HIV-1 MAL sequence.

### 2.2. NP Complex Assembly and Electrophoretic Mobility Shift Assay

Unless stated otherwise, electrophoretic mobility shift assays (EMSA) contained 1 ng/μL NA templates (0.4 nM) in 20 μL and were performed at 37 ${}^{\circ }$C in a binding buffer containing 20 mM Tris-HCl pH 7.4, 1 mM MgCl${}_{2}$, 100 mM NaCl, and 1 mM DTT. NCps were diluted on ice in the reaction buffer. For reactions using NCp15, the buffers were supplemented with 0.1% (*w*/*v*) Tween 20. Reactions were initiated by addition of NC as appropriate and terminated at 30 min. (unless stated otherwise) by chilling the tubes on ice and addition of 10% (*v*/*v*) loading buffer (30% glycerol, 0.01% xylene cyanol, 10 mM Tris-HCl pH 7.4). Samples were then fractionated on 25 cm long, 1% (*w*/*v*) agarose gels (SeaKem LE Agarose, Lonza, Switzerland) in 0.5× TBE. Gels were run in a Sub-Cell GT cuvette (BioRad, USA) for 17–18 h at room temperature at 3 V/cm, stained with SybrGold (Invitrogen, USA), and scanned for fluorescence using a Typhoon 8600 (GE Healthcare, USA). All experiments were performed at least in triplicate.

### 2.3. Dynamic Light Scattering

Dynamic light scattering (DLS) measurements were carried out in 20 mM Tris-Acetate pH 7.4, 50 mM sodium acetate, and 1 mM DTT, using 2.5 ng/μL M13 ssDNA. Experiments were performed with a Zetasizer Nano-ZS (Malvern Panalytical, UK) and high precision cells (QS 3.0 mm, Hellma Analytics, Germany). Measurements were performed at 37 ${}^{\circ }$C, 20 min after addition of the indicated amount of NCp, and analysed by the Dispersion Technology Software provided.

### 2.4. AFM Imaging

NC:NA complexes were assembled under conditions used for EMSA with 1 ng/μL of M13 ssDNA and in a binding solution containing 10 mM TrisAcetate pH 7.0, 50 mM sodium acetate, 2.5 to 5 mM magnesium diacetate, and 0.5 mM TCEP. A freshly cleaved muscovite mica surface was pre-treated for 2 min with a fresh dilution of spermidine (50 μM), extensively rinsed with water and dried under a nitrogen flow [96]. A 5 μL drop of the NP complexes was deposited on the surface and incubated for 3–5 min and dried with nitrogen gas. AFM images were carried out in air with a multimode scanning probe microscope (Bruker, USA) operating with a Nanoscope IIIa or V controller (Bruker, USA) and silicon AC160TS cantilevers (Olympus, Japan) using the tapping mode at their resonant frequency. The scan frequency was typically 1.0 Hz per line and the modulation amplitude was a few nanometers. A second-order polynomial function was used to remove the background with the AFM software (Bruker, USA).

### 2.5. Proteolysis Assays

NC cleavage and SDS-PAGE analysis: A proteolysis assay of NCp15 bound to ssDNA using recombinant PR was reported previously [58]. The assay was optimized in this study to ensure a detailed analysis of the reaction using SDS-PAGE electrophoresis (Supplementary Figure S3a,b). Peptides were quantified by fluorescent staining, which allowed accurate measurements in the 25–500 ng range, in agreement with our NP complex analysis. The standard proteolysis assay contained NCp proteins (6 μM) incubated with NA for 5 min at 37 ${}^{\circ }$C in 10 μL of a PR buffer (MES 50 mM/Tris variable to adjust pH, NaCl 100 mM, DTT 2 mM, BSA 50 μg/ml). Next, PR was added, unless otherwise indicated, at a concentration of 600 nM. Reactions were stopped by addition of an SDS-PAGE loading buffer and heat denaturation (5 min at 95 ${}^{\circ }$C), followed by 1 h incubation at 37 ${}^{\circ }$C in presence of 300 mM Iodoacetamide, which prevented protein oxidation (Supplementary Figure S3a). Samples were separated on 20% acrylamide gels using Tris-Tricine SDS-PAGE in a Hoeffer MiniVE system. After migration at 160 V for 2.5 h, the gels were fixed by 40% ethanol/10% acetic acid for 1 h and stained overnight in 200 mL of Krypton Fluorescent gel stain (Thermo Fisher Scientific, USA) diluted 1/10 in water. Gels were then rinsed with 5% acetic acid and incubated in milliQ water for 30 min before scanning with a Typhoon 8600 imager. Fluorescence counts were quantified using the ImageQuant software (GE Healthcare, USA). Apparent Vmax was measured by dividing the product concentration by the time of incubation with [product]/[S0] product ratio less than 30%. The PR cleavage assay of Figure 1E–F was performed by incubating NCp15 (750 nM) and M13 ssDNA (1 nM) in MES 50 mM/Tris pH 6.25, 100 mM NaCl, 4 mM MgCl${}_{2}$, and 2 mM DTT for 15 min at 37 ${}^{\circ }$C in 50 μL. PR (35 nM) was added, and the cleavage was carried out at the indicated times. Each reaction was stopped by chilling the tubes on ice while a 5 μL drop was used to prepare mica for AFM; 15 μL were loaded on the gel for EMSA, and the remaining 30 μL were used for SDS-PAGE after treating the samples as previously indicated. All experiments were performed at least in triplicate.

FRET assay: The proteolytic activities of PR were determined using the principles of Förster resonance energy transfer (FRET) by cleavage of a fluorogenic peptide substrate DABCYL-γ-abu-Ser-Gln-Asn-Tyr-Pro-Ile-Val-Gln-EDANS (Bachem, Germany) with DABCYL, 4-(4${}^{\prime }$-di- methylaminophenylazo)benzoyl; γ-abu, γ-aminobutyric acid; and EDANS, 5-[(2-aminoethyl)amino] naphthalene-1-sulfonic acid. Incubation of PR with the probe resulted in specific cleavage at the Tyr-Pro bond and a time-dependent increase in fluorescence intensity that is linearly related to the extent of substrate hydrolysis. Kinetic experiments were carried out at 30 ${}^{\circ }$C in 150 μL of the PR buffer (50 mM MES-Tris combination, 0.1–1 M NaCl, pH adjusted between 5 and 7), 5.2 μM of the probe, and 10–50 nM of PR. The probe was first dissolved in DMSO. The final DMSO concentration was kept at 3% (*v*/*v*). Fluorescence intensities were measured in a BMG Fluostar microplate reader. Delay time for the reaction start was calculated as the reaction slope intercept with the *x* axis. All experiments were performed at least in triplicate.

### 2.6. Electron Microscopy of HIV-1 Particles

Maturation mutants: Mutant virions accumulating NCp15 or NCp9 were produced by transfection of mutated pNL4-3 proviral plasmids as described [84]. Plasmids were transfected into HEK 293T cells using Mirus TransIT 293 (Mirus Bio LLC, USA) according to the manufacturer’s instructions. Forty-eight hour culture supernatants were clarified and virions ultracentrifuged and examined by electron microscopy as described previously [97,98]. At least 180 particles were analysed on the criteria that they were enveloped and a contrast was visible inside. Then, the subpopulation of the diffuse cores instead of thin, dark spots was scored with, as discriminating criteria, a diameter equal to or larger than 70% of the internal diameter of the particle.

Viral particles produced from latently infected cells: Latently infected ACH2 cells [99] were grown under standard conditions, were plated onto 10 cm cell culture dishes at densities of 4 × 10${}^{6}$ cells, and incubated with or without a PR inhibitor (10 μM Lopinavir, Sigma). HIV production was activated by adding Vorinostat (10 μM; Sigma-Aldrich, Germany). After 2 days, ACH2 cells were fixed with 2.5% glutaraldehyde, washed, dehydrated, and embedded in epoxy resin according to standard procedures [100]. Electron microscopy images were obtained with a Tecnai Spirit microscope coupled with a 1376 × 1024 pixel CCD camera (FEI, Eindhoven, The Netherlands). We analysed 500 particles attached to the membranes after normal production and 120 after production in presence of Lopinavir (respectively 46.1 and 53% of the total number of detectable particles). Within each attached population, mainly 91% of particles were identifiable, 89.6% containing a dark spot compared to only 1.4% immature for the normal population, while dark spots were not visible after viral production in presence of Lopinavir.

### 2.7. Molecular Dynamics Simulations and Analysis

Molecular dynamics simulations of the NC-SP2 octapeptide were previously performed [101] following a well-established protocol [102], described subsequently. An initial structure was prepared for the NC-SP2 (RQAN-FLGK) octapeptide ligand in apo-form. Atomic coordinates for the octapeptide were extracted from the 1TSU crystal structure [102,103]. The standard AMBER forcefield (ff03) [104] was used to describe all parameters. The system was solvated using atomistic TIP3P water and then electrically neutralized with an ionic concentration of 0.15 M NaCl, resulting in a fully atomistic, explicit solvent system of approximately 14,000 atoms. Conjugate-gradient minimization was performed. The SHAKE algorithm was employed on all atoms covalently bonded to a hydrogen atom. The long range Coulomb interaction was handled using a GPU implementation of the particle mesh Ewald summation method (PME). A non-bonded cut-off distance of 9 Å was used with a switching distance of 7.5 Å. During equilibration the position of all heavy peptide atoms was restrained by a 0.5 kcal/mol/Å${}^{2}$ spring constant for all heavy protein atoms, and the system evolved for 10 ns with a timestep of 4 fs. The temperature was maintained at 300 K using a Langevin thermostat with a low damping constant of 0.1/ps and the pressure maintained at 1 atm for both systems. The system was then equilibrated for 10 ns of unrestrained simulation in the canonical ensemble (NVT) with an integration timestep of 4 fs. The final coordinates were used as input for production simulations. All subsequent simulations were carried out in the NVT ensemble. All production simulations were carried out using ACEMD [105]. An ensemble of 10 × 1 μs production simulations was performed. Coordinate snapshots from production simulations were generated every 10 ps, resulting in an ensemble of 1 × 10${}^{6}$ conformers for subsequent analysis.

In the analysis conducted here, the octapeptide was first relabelled as R52-Q53-A54-N55-F56-L57-G58-K59. The conformer ensemble was then analyzed in a reaction coordinate space consisting of two order parameters: the K59-Q53 C${}_{\alpha }$ distance (d${}_{KQ}$) and K59-F56-Q53 C${}_{\alpha }$ angle ($\theta_{FQK}$). The potential of mean force (PMF) was calculated by binning the ensemble data into microstates corresponding to the given reaction coordinate space and then calculating the mole fraction (ρ) of each microstate using PMF = −k${}_{B}$Tln(ρ), where k${}_{B}$ is the Boltzmann constant and T the temperature (k${}_{B}$T ∼ 0.6 kcal/mol). Corresponding order parameters were calculated for each of the NMR conformers in PDB 1F6U of the NC N-terminus where d${}_{KQ}$ was the K3-Q10 C${}_{\alpha }$ distance and $\theta_{FQK}$, the K3-F6-Q9 C${}_{\alpha }$ angle. Conformers were then aligned to the NC N-terminus from PDB 1F6U by the C${}_{\alpha }$ atoms of K59-R52 mapped to K3-R10 of 1F6U. The C${}_{\alpha }$ RMSD was then calculated as a third-order parameter (d${}_{n}$), its probability density was determined by binning (ρ(d${}_{n}$)), and conformers within the thresholds of 1 Å, 1.5 Å, and 2 Å were extracted and mapped back to the d${}_{KQ}$—$\theta_{FQK}$ reaction coordinate space.

## 3. Results

### 3.1. Cleavage of NCp15 to NCp9 and NCp7 Underpins Weak-Strong-Moderate Quinary Condensate Properties

**Figure 2:**
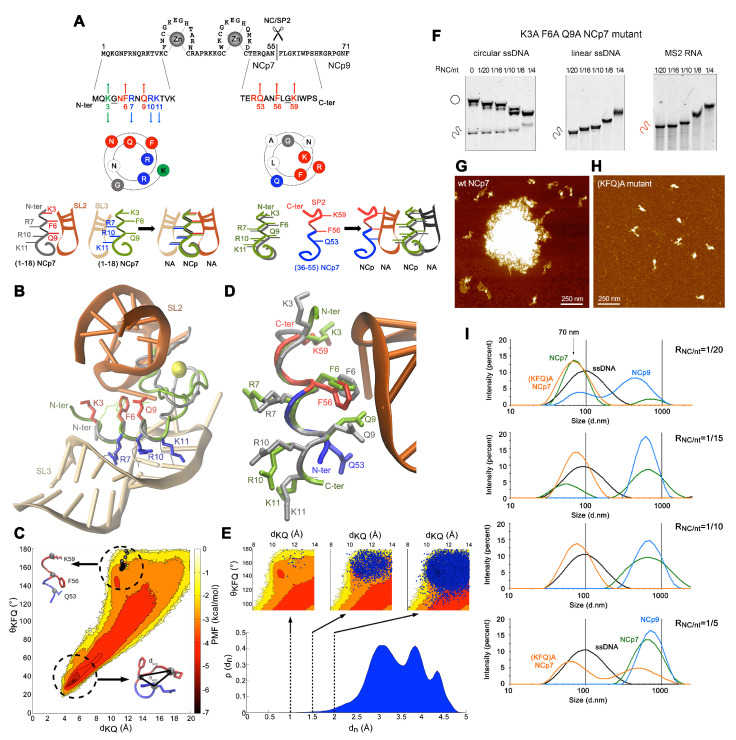
**NC:NA quinary interactions.** (**A**) The N-terminal 3${}_{10}$-helix and the NC-SP2 cleavage site contain DNA strand bridging motifs. The Wenxiang diagram shows the residues of the N-terminal 3${}_{10}$-helix in contact with SL3-RNA (red) or SL2-RNA (blue). The diagram of the 53–60 sequence shows the Q53-F56-G58-K59 motif mirroring the K3-G4-F6-Q9 motif in contact with SL2. The schematics indicate possible NA-NC-NA network assemblies provided by such NA binding domains. (**B**) Superposition of the N-terminal domain backbone of NCp7 in complex with SL3 (PDB ID: 1A1T) and SL2 (PDB ID: 1F6U). (**C**) Potential of mean force (PMF) plot in the 2D reaction coordinate space of the K59-Q53 C_α_ distance (d_*KQ*_) and K59-F56-Q53 C_α_ angle (*θ*_*FQK*_) order parameters. The NC-SP2 apo-octapeptide exhibits substantial flexibility and is energetically dominated (PMF ∼ 5–7 kcal/mol) by a turn-like structure (d_*KQ*_ ∼ 4–6 Å, *θ_KFQ_* ∼ 20°–40°). The conformational ensemble also samples a region (d_*KQ*_ ∼ 10–12 Å, *θ_KFQ_* ∼ 140°–180°) with less frequency (PMF ∼1–3 kcal/mol) that is compatible with a 3_10_ helical structure and where NMR conformers of NCp7 from 1F6U (grey circles) are located. For NMR conformers, d_*KQ*_ corresponds to the K3-Q10 C_α_ distance and *θ*_*FQK*_ to the K3-F6-Q9 C_α_ angle. (**D**) Structure of best-fitting conformer of the R52–K59 segment superimposed head-to-tail with the N-terminal domain of NCp7. (**E**) Probability distribution of the conformer ensemble C_α_ root-mean-squared deviation (RMSD) of the K59-R52 residues aligned to the K3-R10 residues of a single structure of the N-terminal 3_10_ helix in 1F6U. Three conformer sub-populations within an RMSD of 2 Å, 1.5 Å and 1 Å were extracted. Mapping the conformers onto the d_*KQ*_–*θ_KFQ_* order parameters (blue circles) show they occupy the same conformational sub-space. (**F**) Electrophoretic migration in agarose of M13 ssDNA (circular and linear forms) or MS2 RNA in complex with increasing concentrations of a K3AF6AQ9A NCp7 mutant. (**G**,**H**) AFM imaging of wt-NCp7 and K3AF6AQ9A NCp7 mutant incubated with M13 ssDNA in the absence of magnesium. (**I**) DLS analysis of NCp7 (green), NCp9 (blue), and K3AF6AQ9A NCp7 mutant (orange) in complex with M13 ssDNA (black) for increasing NC:nt ratio in the absence of magnesium. Experiments with free and bound-ssDNA are reported on the same graph for clarity.

We first focused on the quinary interactions and the architectural behaviour of NC:NA complexes by combining a repertoire of approaches including an electrophoretic mobility shift assay (EMSA) in agarose gels, SDS-PAGE and atomic force microscopy (AFM) (Figure 1B–F and Figure 2F,H), together with sequence-structure considerations and molecular dynamics simulations (Figure 2A–E) as well as dynamic light scattering (DLS) (Figure 2I).

Examining RNPs with large ssNA templates under increasingly dilute conditions interestingly switched NCp7 binding from NA aggregation (quinary interactions) to intramolecularly-folded NP condensates (Figure 1B). NCp7 binding titrations on a circular M13 ssDNA showed a progressive process of ssDNA migration acceleration in a gel (Figure 1C), seen by AFM as tightly compact NP structures formed of folded DNA strands coated and bridged with protein (Figure 1D). Maximum ssDNA compaction was reached for saturating amounts of one NCp7 over 8–10 nt [77]. Additional protein resulted in the fusion of these NP condensates into very high molecular weight structures that exhibited smearing during electrophoresis.

AFM showed a progressive accumulation of protein clusters covering the lattices, while the branched and secondary structures of the ssDNA appeared melted or absent, and rather bridged into nucleofilament-like structures (Supplementary Figure S2). Omission of magnesium in the buffer (Figure 2G–I) or an excess of NCp7 resulted in the fusion of the individual condensates into huge macrostructures with a spheroid shape comparable with previously described NC:NA aggregates [57,58,73]. NCp7 mobility was deemed necessary since this fusion was not observed, and condensation was delayed at 4 ${}^{\circ }$C [79,86] (Supplementary Figure S1a). The kinetics of the reaction indicated fast intramolecular condensation and a slow process of NP condensate fusion (Supplementary Figure S1b). Low monovalent salt concentration increased NCp7/ssDNA aggregation, and a strong electrostatic competition was observed with Na${}^{+}$ or Mg${}^{2+}$, as expected [73,106] (Supplementary Figure S1c). Mutations of key aromatic residues, Phe16 and Trp37 (Supplementary Figure S1d,e), demonstrated that ssDNA condensation depends not only on phosphate backbone neutralization but also on base capture by the ZF domain. The apo-protein SSHS NC mutant [85] promoted DNA aggregation without acceleration of DNA mobility as expected for polycation-induced NA aggregation [107]. Finally, Ala substitution of basic residues in the N-terminal domain and the linker demonstrated these residues to be essential for ssDNA condensation, as expected. Strand circularity, e.g., the ssDNA topological constraint, favoured intramolecular ssDNA bridging, whereas intermolecular ssDNA-NC-ssDNA interactions were enhanced with linear M13 ssDNA or MS2 RNA (Supplementary Figure S1f), which demonstrated protein-NA networks involving NA-protein-NA and protein-NA-protein interactions, as proposed previously [108].

The binding of NCp9 yielded fast-migrating NP condensates for the lowest protein concentrations (Figure 1C), indistinguishable by AFM from those formed with NCp7 (Supplementary Figure S2f). However, NCp9-driven NA condensation was seen to be dramatically associated with a huge fusion process by EMSA (Figure 1C), DLS (Figure 2I), and AFM (Figure 1D, Supplementary Figure S2f,g). The linearity of the ssDNA template resulted in a huge aggregation, demonstrating the presence of an additional NA binding site in SP2 reinforcing NA–NC–NA networks (Figure 2). In contrast to NCp9 or NCp7, reaching a plateau of one NCp15 per 10–12 nt, NCp15 binding to any of the three templates yielded NP complexes of lower gel mobility upon protein addition (Figure 1C), similar to canonical ssDNA binding proteins [96]. AFM visualization showed passive ssDNA coating instead of bridging compaction within individual complexes for limiting NCp15 concentration, which then led to globular structures at saturation (Figure 1D, Supplementary Figure S2h,i). NCp15 and NCp7 retain equivalent net charges [66] (NCp15 pI 9.93; NCp7 pI 9.59). Therefore, NCp15 binding does not actively compact and aggregate ssNA, confirming previous results [57,60]. With free NCp15, p6 has been proposed to bind to the NC domain [60]. Similar to NCp15 from HTLV-1 [109], HIV-1 NCp15 binding to NA might invoke quinary intermolecular p6-NC contacts instead of quinary NA-NC contacts. These interactions may freeze these globular structures and mask or block the NC residues responsible for NA compaction/aggregation. Followed by EMSA, SDS-PAGE, and AFM, the dynamics of quinary NC-NA interactions through the cleavage of M13 ssDNA-bound NCp15 is verified (Figure 1E,F) from weak in the presence of NCp15, to strong with NCp9, and moderate with NCp7. From globular (NCp15), individual NP complexes are progressively converted into intramolecular condensates (NCp7) after an intermediate step of fusion (NCp9).

### 3.2. Transiently Unmasked NC Binding Sites Enable Modulation of NC:NA Molecular Interactions

A superposition of the N-terminal 3${}_{10}$ helix from the NMR structures of NCp7-SL2 and NCp7-SL3 complexes is shown in Figure 2A,B and reveals two slightly different NA backbone binding motifs for this domain, which could be virtually sandwiched between two RNA stems, providing a bridge to form RNA-NC-RNA networks [108]. Three additional basic residues over the sixteen present in SP2 poorly explain the dramatic enhancement of the NA quinary capabilities of NCp9. Examination of the NCp9 primary sequence reveals that the NC-SP2 cleavage site surprisingly contains five of the eight residues of the NCp7 SL2-binding motif Lys-Gly-x-Phe-x-x-Gln-Arg, but oriented in reverse, from the C- to N-terminus (Figure 2A,D).

To determine if conformers of this sequence would be compatible with an NA binding site structurally similar to those of the N-terminal 3${}_{10}$-helix, we performed all-atom molecular dynamics (MD) simulations of an NC-SP2 octapeptide cleavage site. The resulting conformational ensemble was analyzed in terms of the potential of mean force (PMF) in the 2D reaction coordinate space consisting of the K59-Q53 C_α_ distance (d_*KQ*_) and K59-F56-Q53 C_α_ angle (*θ*_*FQK*_) order parameters. The octapeptide samples a varied conformational space (Figure 2C) that is significantly disordered, like other disordered peptide regions in HIV-1 [110,111]. However, it is energetically dominated by a turn-like structure (d_*KQ*_ ∼ 4–6 Å, *θ*_*KFQ*_ ∼ 20°–40°, PMF ∼5–7 kcal/mol) and also less frequently samples a region (d_*KQ*_ ∼ 10–12 Å, *θ*_*KFQ*_ ∼ 140°–180°, PMF ∼ 1–3 kcal/mol) compatible with a 3_10_ helical structure and similar to NMR structures of the corresponding NCp7 motif (Figure 2C,D). Similarly, the majority of conformers in the ensemble exhibit large RMSDs (>2.5 Å) with respect to an NMR structure of NCp7 (Figure 2E). Nonetheless, three extracted conformer sub-populations, within an RMSD of 2 Å, 1.5 Å and 1 Å, occupy the same region of the conformational sub-space (Figure 2E), with a sub-population ensemble frequency of 9.1 × 10^−3^, 1.3 × 10^−3^ and 3 × 10^−5^, respectively. Therefore, conformers in the MD ensemble that are structurally similar to the N-terminal 3_10_ helix of NCp7 match the corresponding region of the characterized conformational space.

The K3A/F6A/Q9A-mutation in NCp7 mostly abrogated ssNA aggregation but maintained ssDNA M13 condensation, suggesting this triad to be mostly involved in quinary interactions stabilizing NA:NC networks (Figure 2F–H). A DLS analysis in low magnesium finally demonstrated an NCp7/NCp9-driven compaction of M13 ssDNA from 100 nm to 70 nm, followed by a massive fusion/aggregation of these complexes (Figure 2I). In contrast, the K3A/F6A/Q9A NCp7 mutant was strongly defective in the fusion/aggregation process. Altogether, these data strongly support a model where the Lys(3/59)-Gly(4/58)-x-Phe(6/56)-x-x Gln(9/53)-Arg(10/52) octad would act in both NCp7 and NCp9 as a quinary interaction module, establishing bridges between NC-NA complexes at NA saturation. These positions are highly conserved amongst all the HIV-1 subtypes, except at position 3 where the conserved K and R residues are found equiprobable.

### 3.3. Quinary Cooperation between NC and RNA Drives PR Sequestration and RNA-Length-Dependent Catalytic Acceleration

**Figure 3:**
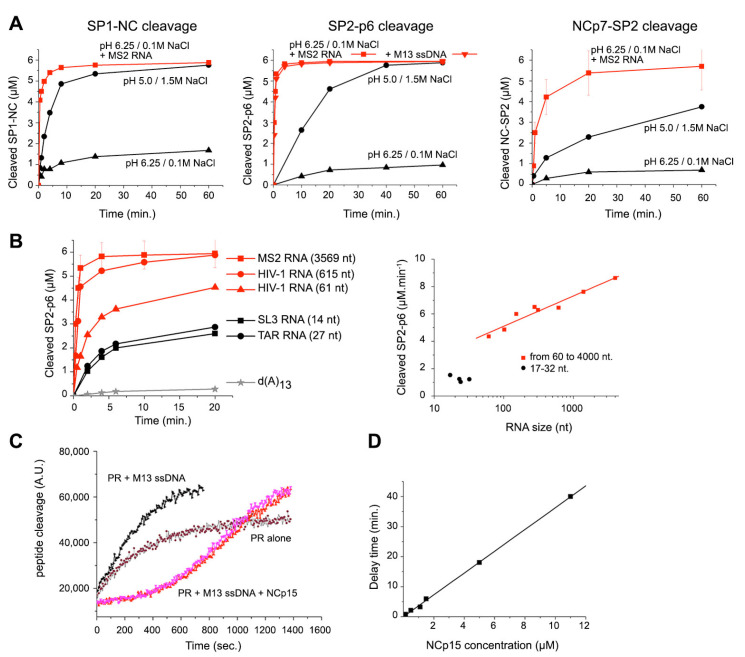
**Nucleocapsid maturation is promoted by NA through sequestration of PR.** (**A**) In vitro proteolysis of SP1-NC, SP2-p6, and NCp7-SP2 cleavage sites by PR in absence (black) or presence (red) of M13 ssDNA or MS2 RNA under unfavourable conditions (pH 6.25 and 0.1 M NaCl). NC concentrations were fixed at 6 μM, PR at 600 nM, and NA at 120 μM (nucleotide), unless otherwise indicated. In absence of NA, PR optimum is found at pH 5.0 in presence of 1.5 M NaCl. The assays, which used peptide fluorescence quantification after SDS-PAGE separation of the cleavage products, are described in Supplementary Figure S3a,b. (**B**) Comparison of NCp15 cleavage by PR in presence of MS2 RNA, NC-specific HIV RNA stem-loops (SL3, TAR), HIV gRNA fragments (1–615, 1–61), and a d(A)13 oligonucleotide, a weak NC substrate. The RNC/nt was fixed at 1/20 for each experiment. NCp15 cleavage rate was calculated as a function of RNA length (SL1 (17 nt), SL2 (23 nt), SL3 (14 nt), SL4 (24 nt), and HIV gRNAs fragments 1–61, 1–102, 1–152, 1–278, 1–311, 1–615, 1–1333, and 1–4001 are reported in the right panel). (**C**) Cleavage of a DABCYL-MA-CA-EDANS peptide probe (5.2 μM) by PR (50 nM) at 30 ${}^{\circ }$C followed by FRET. The reactions were performed with PR alone at pH 5.5 in presence of 0.1 M NaCl, PR and M13 ssDNA (13.4 nM), and PR + M13 ssDNA (13.4 nM) + NCp15 (5 μM). (**D**) Delay of the FRET probe cleavage extrapolated from (C) as a function of M13 ssDNA:NCp15 complex concentration, with RNCp15/nt = 1/20.

Followed by SDS-PAGE under conditions optimized for peptide quantification (Supplementary Figure S3a,b), in vitro processing of the C- and N-terminal extremities of the NC domain in an environment unfavourable for PR dimers (0.1 M NaCl, pH 6.25) reveals a dramatic acceleration of NCp15, NCp9, and NCp7 production in the presence of ssNA templates (Figure 3A, Supplementary Figure S3c–e). A total of 100% of ssDNA- or RNA-bound NCp15 was cleaved in two distinct steps, producing NCp9 and then NCp7 within minutes, confirming a distributive reaction without consecutive cuts upon the same NCp15 copy (Supplementary Figure S3c). Without NA, complete NCp15 cleavage occurred but at a slower rate, only under acidic (pH 5.0) and high salt (1.5 M NaCl) conditions (Supplementary Figure S3d), also concomitantly producing a shorter product (NCp7*). Similar effects were observed with NA for NCp9 cleavage (Supplementary Figure S3e), and the NC-SP2 cleavage appeared 2–3 times slower than that of SP2-p6, either starting from NCp15 or NCp9, but was completed in minutes, much faster than previously shown [68]. MS2 RNA activation also occurs for the SP1-NC site of a GagΔMA protein (CA-SP1-NC-SP2-p6), confirming previous results using a GagΔp6 (MA-CA-SP1-NC-SP2) protein [63] (Supplementary Figure S3f).

We focused on the NC-ssNA NP assemblies and their influence on NCp15 cleavage at pH 6.25 and 0.1 M NaCl. We first compared the influence of a large-scale assembly of NCp15 on M13 ssDNA or MS2 RNA versus stoichiometric complexes formed between NC and a TAR RNA stem-loop (Supplementary Figure S4a). The NA concentrations were varied for a fixed concentration of NCp15. A biphasic effect was observed in the presence of either long ssNA, PR reaching maximal activity when NCp15 saturated the ssNA lattices (Supplementary Figure S4b). A substantial reduction in PR efficacy was observed upon dispersion of NCp15 over the lattice, even though cleavage was maintained at a much higher level than in the absence of NA. In contrast, neither a biphasic effect nor a rapid rate was observed in the presence of the TAR-RNA.

This NA chain-length effect was next followed for NCp15 cleavage, maintaining an equal nt concentration for various HIV-1 RNA stem-loops and fragments from 61 to 615 nt (Figure 3B, Supplementary Figure S4c). A weak NC substrate, d(A)13 oligonucleotide, was ineffective in stimulating PR activity, whereas the TAR and SL3 RNA led to significant but incomplete stimulation of the reaction. Above a critical threshold (∼50 nt-length) and for a pH optimum around 6.3 (Supplementary Figure S4e,f), PR activity scaled non-linearly with RNA length irrespective of biological origin (Figure 3B). Without NA, a 5-minute incubation between NCp15 and PR in 0.1 M NaCl at pH 6.25 resulted in no cleavage, while the addition of MS2 RNA immediately boosted the reaction (Supplementary Figure S4d). Diluting PR for fixed NC:PR (10:1) and NA:NC (20 nt.:1) ratios revealed a process resistant to dilution for an NA larger than 615 nt (T1/2 from 0.15 to 1 min), whereas a strong rate decrease was observed for TAR or cTAR structures (T1/2 extrapolated to 3 h when considering the first quarter of the reaction; Supplementary Figure S4e). A regular decrease at pH 5.0 and 1.5 M NaCl was observed in the absence of NA (T1/2 from 4 to 25 min).

These large NA chains greatly stimulated NCp15 cleavage at 0.1 M salt, with a remarkable pH optimum between 6.0 and 6.5 (Supplementary Figure S4f). The NCp7* extra-cleavage, previously described, corresponds to a site at position 49–50 as a result of ZF destabilization at low pH [112]. This product was examined in two NCp15 cleavage mutants (Supplementary Figure S4g) and was due to the cumulative effects of both MS2 RNA and acidic pH, which clearly “overcut” the NC domain at pH 5.4. A mildly acidic pH therefore appears beneficial in reducing irregular cleavages of NCp7 upon high PR turnover. With such optimized conditions that satisfy both PR efficiency and NC folding while restricting NCp7 cryptic site cleavages, we confirmed the RNA length effect for the NCp9-to-NCp7 reaction (Supplementary Figure S4h,i), leading to a maximal observed rate close to the T1/2-value of the NCp15-to-NCp9 reaction rate, under conditions where NCp9 and NA appeared strongly aggregated (see Figure 1).

Finally, in the presence of 1:1 NC ligands (TAR, cTAR, SL3), bound NCp15 appears almost individually distributed in the reaction mix and allows a reaction-diffusion mechanism that accelerates PR turnover, but under conditions where native PR is much less stable. Any substance able to increase the local concentration of either the substrate or the enzyme, or both, drives the reaction in the forward direction, enhancing enzyme turnover. As such, ssNA length-dependent activation engages an NC crowding effect with NCp15 molecules coating the NA lattice and forming clusters, trapping PR independently of the NP complex concentration. These NA-scaffolded clusters allow a faster PR turnover, making both SP2-p6 and NC-SP2 cleavages much more efficient. In other words, the NA quinary capabilities of the NC domain induce an RNA-driven sequestration effect on PR.

To better understand this sequestration phenomenon, we used a FRET-based assay that measures the cleavage rate of an MA-CA octapeptide probe in the presence of NA and/or NCp15. The assay firstly confirmed the reduction of PR activity upon pH increase and salt dilution (Supplementary Figure S5a,b). The M13 ssDNA appeared as an effective substitute to high salt and boosted PR activity by a factor of 10 at pH 5.0, three times greater than in the presence of 1.5 M NaCl. A similar effect also occurred at pH 6.25, although PR activity was strongly attenuated. These results confirmed that non-specific PR-NA interactions result in enzyme activation [64]. Adding an equimolar amount of NCp15 at pH 5.5 did not affect the reaction. In contrast, the addition of NCp15 bound to ssDNA resulted in a total inhibition of the octapeptide cleavage for ∼5 min, before reaching a velocity analogous to that measured in the presence of ssDNA alone (Figure 3C, Supplementary Figure S5c). PR is thus sequestered into the NP complex and completes NCp15 processing prior to cleaving the MA-CA peptide at a rate comparable with ssDNA alone, the delay time being directly proportional to the NCp15 concentration with a fixed NCp15:ssDNA ratio (Figure 3D).

**Figure 4:**
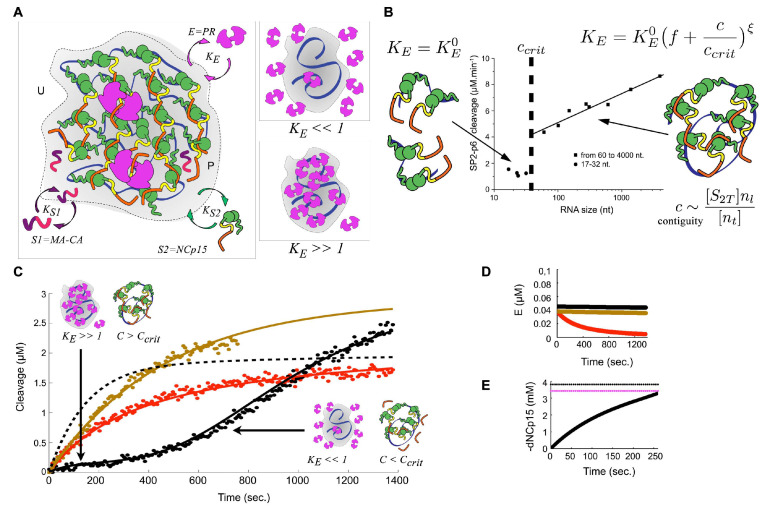
**Theoretical model of RNP-modulated enzyme-substrate reaction kinetics.** (**A**) Reaction rate is governed by a combination of effective concentrations of enzyme (E) and two substrates (S1 and S2) in each of two volume domains, pervaded (P) and unpervaded (U) by NA, as well as the absorption equilibrium for different species. S1 is a competitive substrate, S2 is a component of the RNP. For K${}_{E}$≫ 1, E (in this case, PR) is sequestered into the NA-pervaded volume, whilst for K${}_{E}$≪ 1, its pervaded concentration is reduced. (**B**) A one-substrate rate model (S2 only) is fit to experimental data to determine non-linear K${}_{E}$-dependence (exponent ξ) on the contiguous number *c* of S2 (in this case, NCp15) molecules bound per NA, when above a critical threshold, $c_{crit}$. (**C**) Fitted two-substrate kinetic model of NCp15 and competitive substrate (MA-CA) with NA (black), MA-CA only without NA (red) and MA-CA only with NA (brown) data. For the NCp15-containing system, the early reaction is dominated by high contiguity (*c* > $c_{crit}$, K${}_{E}$≫ 1), inducing enzyme (PR) sequestration. This effect dissipates upon processing (*c* < $c_{crit}$, K${}_{E}$≪ 1). A total of 90% of NCp15 cleavage (dashed black line) is calculated from the model to occur after ∼400 s. (**D**) Differential enzyme decay: The presence of NA stabilizes the PR dimer (black and brown); in the NA-absent reaction, PR decays in the model with an experimental half-life (black). (**E**) Scaling to in virio conditions yields 90% (dotted purple line) NCp15 processing within 260 s (solid black line). The dotted black line represents the 100% processing level.

These data were interpreted by devising a theoretical model of RNP-modulated enzyme-substrate reaction kinetics (Supplementary Note, Figure S4), which partitions the reaction volume between distinct regions that are either occupied (pervaded, P) or unoccupied (unpervaded, U) by NA, and with fractional volumes $\alpha_{p}$ and $\alpha_{u}$ = $\alpha_{p}$− 1, respectively. Reacting species (substrates S1: MA-CA and S2: NCp15; enzyme E: PR) exhibit equilibrium absorption (with equilibrium constants K${}_{S1}$, K${}_{S2}$ and K${}_{E}$, respectively) between these regions due to non-sequence-specific NA-binding (Figure 4A).

Our model builds on a Michaelis–Menten reaction kinetics approach that follows the reactions in both the NA-pervaded and NA-unpervaded volumes individually, whilst relating the transmission of each species across the two domains. Concentrations of each species within each region are derived in terms of the fractional volume, where

(1) $$\alpha_{p}=\kappa N_{A}\left[n_{t}\right]{\left(n_{l}\right)}^{1/2}{\left(\frac{l_{0}l_{p}}{3}\right)}^{3/2},$$

is derived from polymer physics considerations regarding the radius of gyration of a nucleic acid chain ($R_{g}∼{\left(\frac{n_{l}l_{0}l_{p}}{3}\right)}^{1/2}$) [113], and where κ is a volumetric constant, $N_{A}$ is Avogadro’s constant, [$n_{t}$] is the total nucleotide concentration, $n_{l}$ is the number of nucleotides on each NA chain, $l_{0}$ is the length of a single nucleotide, and $l_{p}$ is the NA persistence length. In our model, the enzyme escape rate depends on NCp15 contiguity—the contiguous number (*c*∼ [$S_{2T}$]$n_{l}$/[$n_{t}$]) of NCp15 molecules bound per NA (where [$S_{2T}$], in this case, is [NCp15]—the total concentration of NCp15) and not just chain length or the NC-NA loading ratio alone.

The observed phase transition is consistent with a minimum critical contiguity threshold ($c_{crit}$) required to alter the enzyme escape rate. Therefore, in our model, below this threshold, the enzyme absorption equilibrium is assigned as invariant ($K_{E}^{0}$) and, above the threshold, it is assigned as being non-linearly dependent on *c*, with exponent ξ and constant *f*, such that

(2) $$K_{E}=K_{E}^{0}{\left(f+\frac{c}{c_{crit}}\right)}^{\xi }.$$

Therefore, we describe contiguity as explicitly length dependent: fitting a one-substrate rate model onto the experimental data (Figure 4B) yields the non-linear dependence of K${}_{E}$ on contiguity with exponent ξ∼0.4. and $c_{crit}$∼3. Subsequently, we implement a two-substrate kinetic model that includes a competitive substrate (Figure 4C, Supplementary Table S1, Supplementary Note) and incorporates the effects of differential enzyme decay (Figure 4D). Our model is fitted to, and is compatible with, the observed sequestration effect (Figure 3C and Figure 4C), giving ξ∼1.2 and $c_{crit}$∼3. Reduced sequestration is thus due to reduction in the NCp15 contiguity across the time-course of the reaction—initially the enzyme is absorbed into the RNP (K${}_{E}$≫ 1), and after significant processing, its absorption, and thus sequestration, is reduced (K${}_{E}$≪ 1). Although the experimental data track the competitive cleavage of MA-CA, they do not provide a direct handle on NCp15 cleavage. Nonetheless, the model can calculate NCp15 cleavage directly (dashed black line in Figure 4C), predicting that NCp15 processing is 90% complete after ∼400 s in the experimental assay. Furthermore, when scaled to in virio concentrations of the enzyme and substrate, as well as increased NA length, it predicts a core condensation time of within ∼5 min (Figure 4E). Our model shows that local crowding within the RNP induces cumulative non-linear effects on non-specific enzyme binding. The absorption equilibrium constant itself depends on this local environment, consistent with quinary interactions between PR, RNA, and NCp15 [30].

### 3.4. Condensate-Driven Accelerated PR Processing Temporally Couples Budding to Maturation

**Figure 5:**
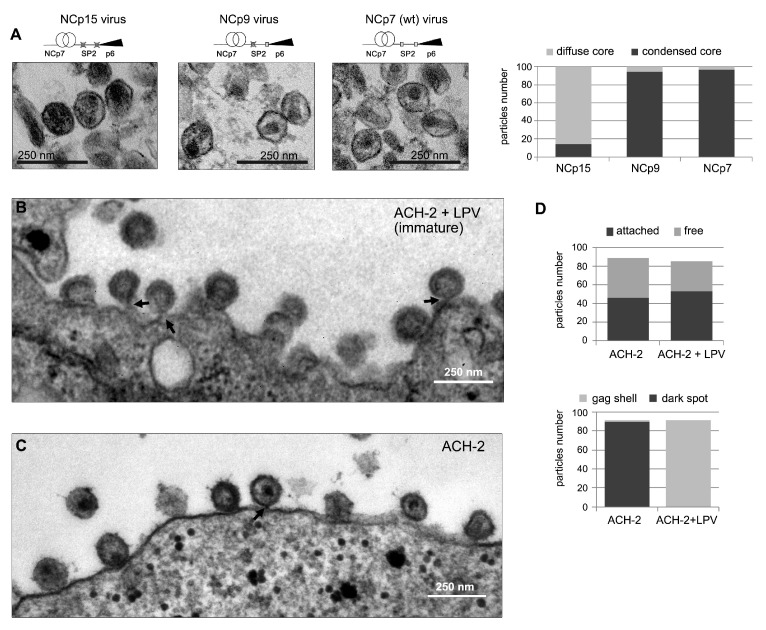
**Nucleocapsid condensation within HIV-1 particles depends on NCp15 processing and is detectable in membrane-attached particles.** (**A**) TEM images of purified HIV-1 NL4-3 virions accumulating NCp15 (uncleavable p6 and SP2 sites), NCp9 (uncleavable SP2), or wt-NCp7. NCp15-containing particles present defects in nucleocapsid condensation, while NCp9- and NCp7-containing viruses show correct core condensation into an electron-dense dark spot. Quantitation was done for 180 counted particles. (**B**,**C**) TEM images of latently infected ACH-2 cells producing viral particles at the plasma membrane after 48 h activation by Vorinostat. The majority of membrane-attached HIV-1 particles produced by latently infected ACH-2 cells are immature particles in the presence of LPV, a PR inhibitor (**B**). In the absence of LPV, the particles contain an electron-dense dark spot indicative of nucleocapsid condensation (**C**). Bottlenecks characterizing budding intermediates are pointed to by arrows. (**D**) Quantitation of attached and free particles (top) and particles containing a condensed RNP (bottom), as noted by a dark spot, in the presence or absence of LPV. Counting was performed for 200 particles for LPV-treated ACH2 cells and 500 particles for non-treated ACH2 cells.

In order to approach this process of RNP condensation in virio, we finally compared by TEM the core content of HIV-1 NL4-3 virus particles assembled with Pr55Gag containing uncleavable NC-SP2 or NC-SP2-p6 sites, thus accumulating NCp9 and NCp15, respectively [84] (Figure 5A and Supplementary Figure S6a).

More than 90% of both NCp9- and NCp7-containing viruses display a morphologically conical capsid encasing an electron-dark spot corresponding to a condensed RNP. In contrast, more than 80% of the NCp15-containing viruses display electron-dark diffuse cores. This demonstrates that the strong-quinary NCp9 intermediate actively triggers nucleocapsid condensation, thus reducing the occupied volume and facilitating capsid rearrangement. We next imaged plasma membrane-attached particles of HIV-1 virus produced from latently-infected ACH2 cells. Washing the cell suspension before fixation enriched the proportion of attached particles engaged in budding. In the presence of a PR inhibitor, all membrane-attached particles appeared immature with a typical electron-dense Gag shell and a bottleneck that characterized budding intermediates (Figure 5B,D). Without an inhibitor, most of the attached particles exhibited a dark spot and a closed envelope (Figure 5C,D). Therefore, the maturation step involving strong-quinary NCp9 occurs visibly in a time frame consistent with both the end of budding [114,115,116] and our kinetic model: budding and maturation appear temporally coupled.

## 4. Discussion and Conclusions

**Figure 6:**
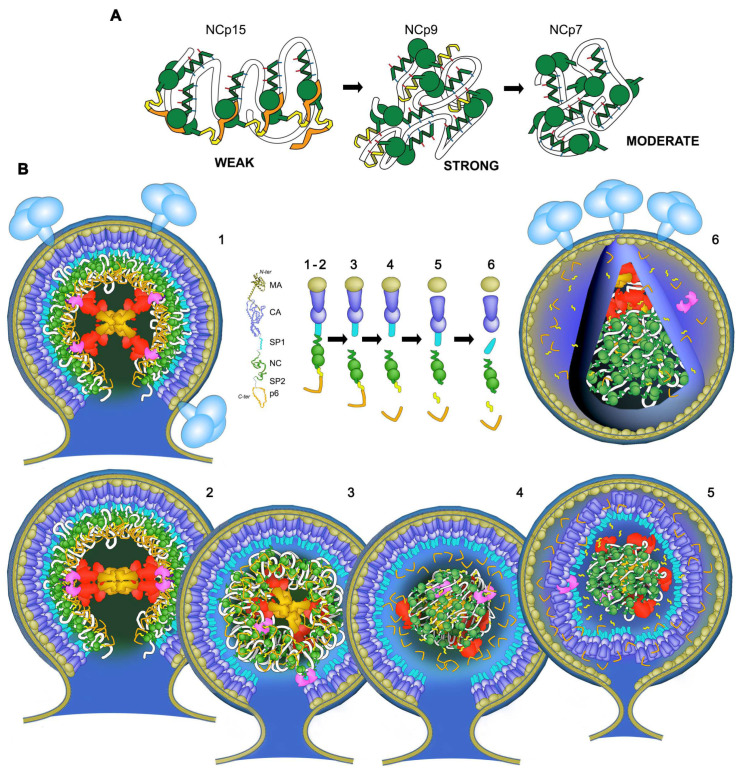
**Nucleocapsid condensation, quinary interactions and WSM transition of the nucleocapsid during HIV maturation**. (**A**) Weak-strong-moderate (WSM) quinary properties of NC proteins with RNA (white) throughout virus maturation. p6 (orange)-NCp7 (green) contacts assemble NCp15 networks upon the RNA, blocking exposed residues engaged in NCp9 or NCp7: NA contacts. Cleavage of p6 unmasks the NCp9 domain, and the NCp7-SP2 (yellow) domain engages NC:NA:NC and NA:NC:NA networks favouring strong RNA-mediated quinary interactions and RNP granulation. Cleavage of SP2 removes one “K/F/Q” NA binding patch (see Figure 2A), which reduces the quinary network between RNA and NCp7. (**B**) Nucleocapsid WSM transition in the context of a virus particle. (**1**) Virus particles at the plasma membrane bud from self-assembly of Gag and GagPol on gRNA, with the NC domain as the RNA binder. The gRNA is shown in white within the layer of NC domains, assembled as bundles of six at the base of Gag hexamers. (**2**) The dimerization of GagPol self-activates PR (pink) and initiates maturation. (**3**) The SP1-NC site is cleaved, liberating the gRNA:NCp15 RNP, which in turns sequesters PR. (**4**) Rapid cleavage of NCp15 into NCp9, which liberates p6, unlocks the strong quinary properties of NCp9. This quickly compacts the gRNA and favours viral budding. gRNA condensation allows internal reorganization of RT (red) and IN (gold), self-assembly of the conical capsid after separation of MA from CA-SP1, and maturation of CA-SP1, while NCp9 are matured to NCp7 in (**5** and **6**).

We describe in this study HIV-1 nucleocapsid maturation as a dynamic RNA granule processing phenomenon, involving differential RNA binding activities of the NC domain that are dependent on processing state. Weak NC-RNA contacts fit with the concept of quinary interactions [28] that lead to gRNA condensation in the context of RNA-directed phase separation [25]. We propose that this RNP follows a dynamic weak-strong-moderate (WSM) quinary model resulting in granular phase-separated RNP condensation (Figure 6) with a distributive three-step processing mechanism in the order of SP1-NC, SP2-p6, and NC-SP2. Each step alters the NC-RNA interaction strength within the confined phase.

The variations in condensing the RNA (in vitro condensation plus aggregation) therefore appear directly linked to both the number of amino acid residues weakly contacting NA chains and the consequent spatial separation within the porous RNP network across different processing states. These contacts are severely limited in NCp15 due to p6 interfering with NC-SP2 NA binding [60,66] and/or competing with the NA for binding to the NC ZF core [76], whilst at the same time p6 may confer additional spacing between RNP components. This is compatible with a biophysical sticker-spacer model that describes biomolecular condensate formation [36]. We also propose that in addition to the polycationic nature of the NC domain [72,77,79,109], two motifs, one in the N-terminal 3${}_{10}$-helix and the other an inverted motif in the NC-SP2 junction, are responsible for NC-NA-NC and NA-NC-NA networks providing a source of quinary interactions. Mutational analyses of these two motifs in future studies may shed further light on the extent of their role in forming such interaction networks. In the crowded in virio environment at neutral or mildly acidic pH, our model also involves quinary PR sequestration by the RNP, which dramatically enhances the global efficiency of the sequential cleavage. These findings are consistent with recent observations that HIV-1 and, more broadly, that retroviral NC can phase-separate in the intracellular environment [55].

Our data confirm, first, that RNA-bound NCp15 avoids strong RNP condensation within the NCp15-gRNA intermediate assembly. The intrinsically disordered p6 likely directs a quinary RNA-NCp15 network via NC:p6 intermolecular contacts that weaken quinary RNA-NC interactions whilst maintaining spatial separation of nearby RNP regions. Such an assembly is deficient in actively aggregating within the viral core, while it may allow the ∼60 PR available in the particle to efficiently access the ∼2400 SP2-p6 cleavage sites and to jump from site to site within close distances (Figure 6). PR previously was shown to be activated by interacting with RNA [64]. We demonstrate here that a faster PR turnover is driven by RNA-clustered NCp15 in an RNP-length-dependent manner where the enzyme is sequestrated by the RNP, enhancing its local concentration and allowing it to efficiently cleave the SP2-p6 octapeptide sites. Our observation of sequestration is consistent with previous studies that found PR packaged inside mature HIV-1 cores [117].

We suggest the mechanistic origin of this sequestration to be the dynamic formation of a network of quinary interactions, derived from multiple, weakly attractive ternary contacts of PR-(SP2-p6)-RNA that together drive the absorption of PR into the porous condensate. Whilst the SP2-p6 octapeptide does not directly appear in contact with RNA, the exposed PR basic residues proposed to bind to RNA [64] may allow this ternary contact, whereas RNA-bound NCp15 may expose its SP2-p6 cleavage site to RNA-bound PR with the NC-SP2 site being inaccessible. One quinary effect would be that progressive cleavage of the SP2-p6 sites within the RNP results in the loss of PR-(SP2-p6)-RNA ternary contacts, driving a reduction in enzyme absorption equilibrium and shifting the balance of PR within the RNP.

An additional mechanism for enhanced PR activity of NCp15 processing may be a directed, rather than diffusive, propagation of PR following the cleavage of an SP2-p6 site—also consistent with RNP-length-dependent acceleration. The inaccessibility of NC-SP2 implies a discrimination process that favours trans PR transfer to neighbouring NCp15 molecules rather than cis sliding along the NCp9 moiety to cleave the RNA-bound NC-SP2 site, even though only 16 aa sequentially separate the two sites. Cleaved p6, already in contact with a neighbouring NC domain as well as PR-RNA contacts, possibly directs the trans event. Both effects are complementary to each other and qualitatively consistent with our model. Indeed, kinetic characterisation of structural transitions between conformations in the PR show a sub-μs timescale adoption of a wide-open state [118]. This state appears excessively open for substrate binding. Thus it may play a role in facilitating directed PR motion through the RNP condensate via the above-mentioned contacts.

When NCp9 forms, the NC-SP2 octapeptide site should bind more easily to RNA due to p6 separation, promoting both the PR recruitment for the NC-SP2 cleavage and the RNA condensation in concert with the other critical NC residues. Again, a ternary PR-NCp9-RNA complex may be required, as the free NCp9 appeared here as a poor substrate for PR, confirming previous results [68]. We propose that the NC-SP2 site is in direct contact with RNA by way of the QxxFxxK triad. The critical NC-SP2 octapeptide also binds within the PR active site for cleavage: this cleavage should require displacement of the bound RNA upon the surface of PR, allowing a quinary contact between RNA and PR. In addition, we also observe the RNP-length-dependent enhancement of NCp9 cleavage, in part suggesting a directed propagation mechanism whereby PR transfers directly from cleaved NCp9 (thus NCp7) to neighbouring NCp9. RNA condensation does not seem to provide any critical physical barrier to PR as NCp9 processing appears quite similar whether the reaction starts from NCp15 or NCp9 (Figure 3A). It is possible then that even a reduced population of PR within the RNP, due to a reduction in absorption equilibrium, may out-compete NCp9 cleavage compared to the absence of RNA. Whilst our theoretical model addresses length-dependent acceleration due to sequestration, it does not explicitly incorporate directed propagation of PR. Future studies and models that dissect both mechanisms may provide further insight into the degree to which each mechanism is at work across the distinct stages of maturation. Nonetheless, taken together, our study suggests the emergent effect is a perfect fit for ordered turnover by PR from site to site with two distinct waves, NCp15-to-NCp9 followed by NCp9-to-NCp7.

Whilst RNA and NC are dispensable for HIV-1 capsid assembly [119], integrative biochemical reconstitution studies have shown that RNA and other cofactors play important synergistic roles in HIV-1 assembly kinetics [120]. In our HIV-1 model, the granulation dynamics shown here opens new avenues at the mesoscopic scale to better understand how the surrounding capsid progresses towards a conical reassembly and what the implication and relocation of RT and IN proteins are. While both mature during GagPol processing, IN has been shown recently to be a key actor in properly coordinating RNP granular condensation and capsid reassembly within the viral particle [121,122,123]. These dynamics also offer new schemes to revisit the proposed implications of HIV-1 Nef, Vif, Tat, and Vpr auxiliary proteins within the design of an infectious particle [124]. Our findings suggest tight temporal control of GagPol incorporation and PR auto-processing supported by the NC domain in GagPol during particle formation [125,126]: this domain should help to position GagPol in the Gag assembly, facilitating the processed PR directed by the gRNA to digest the Gag-domains NC, SP2, and p6. As highlighted by an independent study looking for the production of GagPol VLPs without any viral accessory genes [127], the concomitant interactions of the exposed p6 domains with the ESCRT components, ALIX, and more especially TSG101 [82,128,129] tightly coordinate the complete processing of Gag within just a few minutes of particle release. This is in accordance with what we show here, namely, the exclusive release and processing of mature HIV-1 particles, including a condensed nucleocapsid, accelerated to the same time frame due to a phase-separated effect.

Quinary interactions undoubtedly offer a missing link between molecular and cellular biology [17,25,28,29] both in terms of fundamental understanding and therapeutic application. The quinary aspect of NC-RNA interactions regulating a biomolecular condensate (BC), shown here, provides new perspectives for pharmacological targeting during particle production [130]. Finally, our HIV-1 model adds a novel dimension to the study of BCs and liquid–liquid phase separation (LLPS). Not only does it suggest concentration-driven accelerated enzyme activity in such BCs, but also that the cooperation of RNA, RNA-binding proteins, and an embedded proteolytic machinery can create a scaffolding role for crowded architectures that dynamically regulate their own condensation through the adjustment of quinary interactions [25,29,131]. Given that RNA-containing membraneless compartments have been linked to origins of life chemistry [132], such dynamic condensate regulation mechanisms may be universal properties of RNPs and, moreover, may have emerged early in the evolution of life.

## Supplementary Materials

The following are available online: https://www.mdpi.com/article/10.3390/v13112312/s1, Supplementary Figure S1: Large-scale NA binding of NC proteins followed by EMSA. Supplementary Figure S2: Binding, condensation, or aggregation of M13 ssDNA by NC followed by AFM. Supplementary Figure S3: The PR-driven cleavage of NCp in vitro is strongly activated by NA. Supplementary Figure S4: PR activation of NC proteolysis is modulated by NA length and NC:NAinteractions. Supplementary Figure S5: PR is sequestered by the NCp15:ssDNA NP complex. Supplementary Figure S6: Nucleocapsid condensation is concomitant with budding. Supplementary Table S1: Parameters for RNP-modulated two-substrate kinetic model. Supplementary Note: A theoretical model of RNP-modulated enzyme-substrate reaction kinetics. 1: Theoretical development. 2: Computational implementation and model parameters. 3: Analysis of model features. Supplementary Appendix.

## Abbreviations

The following abbreviations are used in this manuscript:

HIV	Human immunodeficiency virus
RNA	Ribonucleic acid
